# Improved Water-Tree Resistances of SEBS/PP Semi-Crystalline Composites under Crystallization Modifications

**DOI:** 10.3390/molecules25163669

**Published:** 2020-08-12

**Authors:** Jun-Qi Chen, Xuan Wang, Wei-Feng Sun, Hong Zhao

**Affiliations:** Key Laboratory of Engineering Dielectrics and Its Application, Ministry of Education, School of Electrical and Electronic Engineering, Harbin University of Science and Technology, Harbin 150080, China; chenjunqi@hrbust.edu.cn (J.-Q.C.); topix_xuan@sina.com (X.W.)

**Keywords:** polypropylene composite, water blade electrode, dynamic thermomechanical analysis, crystallization kinetics

## Abstract

Water-tree resistances of styrene block copolymer/polypropylene (SEBS/PP) composites are investigated by characterizing crystallization structures in correlation with the dynamic mechanical properties to elucidate the micro-structure mechanism of improving insulation performances, in which the accelerated aging experiments of water trees are performed with water-knife electrodes. The water-tree morphology in spherulites, melt-crystallization characteristics and lamella structures of the composite materials are observed and analyzed by polarizing microscopy (PLM), differential scanning calorimetry (DSC) and scanning electron microscopy (SEM), respectively. Dynamic relaxation and stress-strain characteristics are specifically studied by means of a dynamic thermomechanical analyzer (DMA) and electronic tension machine, respectively. No water-tree aging occurs in both the highly crystalline PP and the noncrystalline SEBS elastomer, while the water trees arising in SEBS/PP composites still has a significantly lower size than that in low-density polyethylene (LDPE). Compared with LDPE, the PP matrix of the SEBS/PP composite represent a higher crystallinity with a larger crystallization size in consistence with its higher mechanical strength and lower dynamic relaxation loss. SEBS molecules agglomerate as a “island” phase, and PP molecules crystallize into thin and short lamellae in composites, leading to the blurred spherulite boundary and the appreciable slips between lamellae under external force. The high crystallinity of the PP matrix and the strong resistance to slips between lamellae in the SEBS/PP composite essentially account for the remarkable inhibition on water-tree growth.

## 1. Introduction

Green initiative and recyclable thermoplastic materials are now promised in advance to be applied for the extruded plastic insulated power cable that can acquire excellent electrical and mechanical properties and good processing performances [1,2], in which polypropylene (PP) represents multiple high performances simultaneously in mechanical strength, heat resistance and electrical insulation [3,4]. Since 2002, when the world’s first 0.6 kV and 22 kV PP cables were produced by blending polyethylene (PE) and antioxidants into a syndiotactic PP (SPP) matrix for cable main insulation, it has been suggested that the alternative current (AC) breakdown strength and dielectric loss of PP material are well-qualified for manufacturing insulated cables in future. Whereas the poor tenacity of PP materials at low temperatures restrains the practical applications in cable major insulation, for which an appropriate quantity of elastomer such as styrene-ethylene/butylene-styrene triblock copolymer (SEBS) or ethylene octene copolymer (POE) is required to be blended into the PP matrix for improving the tenacity. It has been verified that the dielectric breakdown strength of isotactic PP (iPP) will be well-retained when the SEBS has been blended for increasing the flexibility. In the future, polypropylene is a prospective to be widely used for the main insulation of power cables.

A water tree refers to the dendritic discharge trace produced in the polymer dielectrics with water impurities under an electric field. In the inevitable humid environment, water will gradually be absorbed into the insulation layer of the power cable and form a water tree under the AC electric field to cause insulation degradation and dielectric failure, which generally occurs in medium-voltage power cables [5,6,7,8]. It has been substantially demonstrated from sufficient experiments that the inception and growth of water trees do not rely on external factors, such as electric field strength and frequency, aging time and ambient temperature but, also, are affected by internal factors such as crystallization morphology, type and the content of additives in the insulating materials [9,10,11,12]. The water tree will not only lead to the deterioration of the mechanical and dielectric properties of the power cable insulation but, also, develop into an electric tree structure, which will greatly decrease the service life and operation reliability of the power cable. In order to effectively improve the water-tree resistance of the cable insulation, the repairing-fluid injection and nanodielectric technologies are employed to inhibit or alleviate the insulation aging from water-tree growth [13,14,15]. However, at present, the researches concerning the water-tree aging of recyclable thermoplastic materials are primarily focused on low density polyethylene (LDPE), which has demonstrated that water-tree aging is not preferred for polymer materials with high crystallinity [5]. Since the micro-structures of PP and SEBS/PP are different from LDPE, it is reasonably suggested that they will represent an appreciable discrepancy in water resistance.

In this paper, the water-tree resistances of the polypropylene-elastomer blends are studied for further engineering applications. The SEBS/PP composites are prepared by the melt-blending method for the water-tree experiments with a water blade electrode, in comparison to the low-density polyethylene (LDPE) used for fabricating a traditional power cable. The most widely studied iPP and the SEBS with a certain dielectric strength are adopted respectively for the PP matrix and elastomer additive to achieve the composite materials with excellent electrical properties for practical engineering applications [16,17]. The crystallization characteristics and mechanical properties of the SEBS/PP composites and LDPE are investigated to elucidate the intrinsic mechanism of the water-tree formations correlated with crystallization morphology.

## 2. Results and Discussion

### 2.1. Water-Tree Morphology and SEBS Dispersion

**Figure 1:**
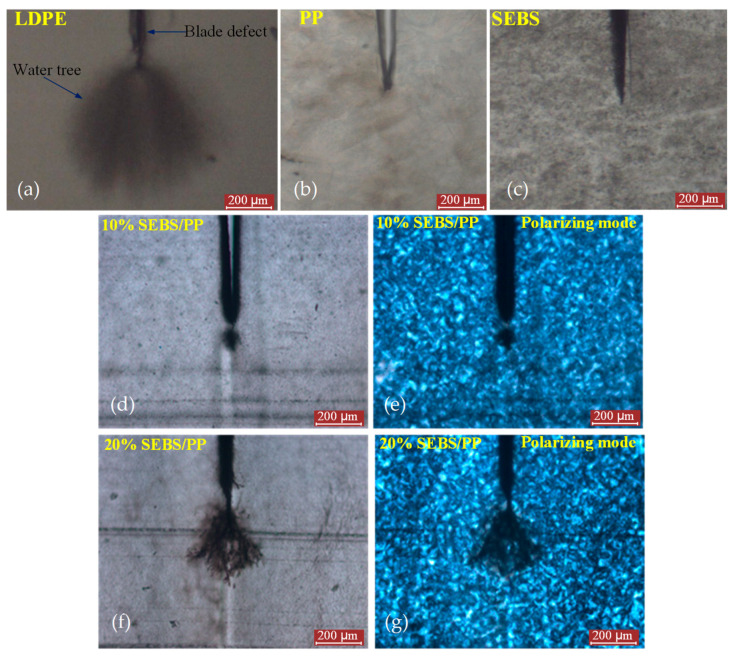
The water-tree morphology photographs of (**a**) low-density polyethylene (LDPE), (**b**) polypropylene (PP), (**c**) styrene-ethylene/butylene-styrene triblock copolymer (SEBS), (**d**–**g**) SEBS/PP composites observed with the optical microscope in normal mode (**d**,**f**) and polarizing mode (**e**,**g**).

**Figure 2:**
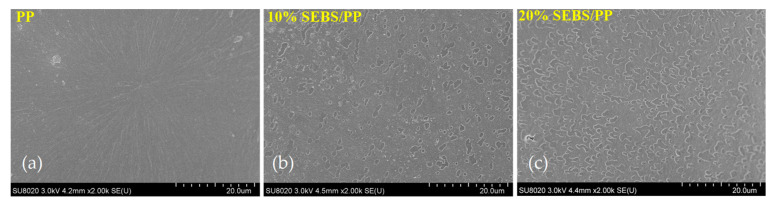
SEM images of (**a**) PP, (**b**) 10%SEBS/PP composite and (**c**) 10%SEBS/PP composite.

The representative morphologies of water trees and spherulites from the observations of optical microscopy and polarizing microscopy (PLM) are shown in Figure 1, with the regions of the water trees, water-blade breach and spherulites being individually indicated with labels. SEBS/PP composites represent a higher water-resistance, as shown by the evidently smaller tree size than that of LDPE. Even though no observable water trees arise in the pure materials of PP and SEBS after the water tree-aging experiments, the SEBS/PP composites show a substantial morphology of the water trees essentially existing in amorphous regions between spherulites, implying that SEBS fillers have introduced void structures into the PP matrix for accommodating water accumulations. It is clearly observed from Figure 2 that the SEBS “island” phases are uniformly dispersed in the PP matrix. The dispersion morphologies of SEBS in the PP matrix further reveal that polystyrene fragments in SEBS molecules lead to a preferential structure of the amorphous morphology that is incompatible with PP crystallization. Therefore, appreciable “voids” appear after SEBS is etched out in SEBS/PP composites, which become larger for higher SEBS contents and indicate the islands of the SEBS amorphous phase being uniformly distributed in the crystalline PP matrix.

### 2.2. Crystallization Characteristics

**Figure 3:**
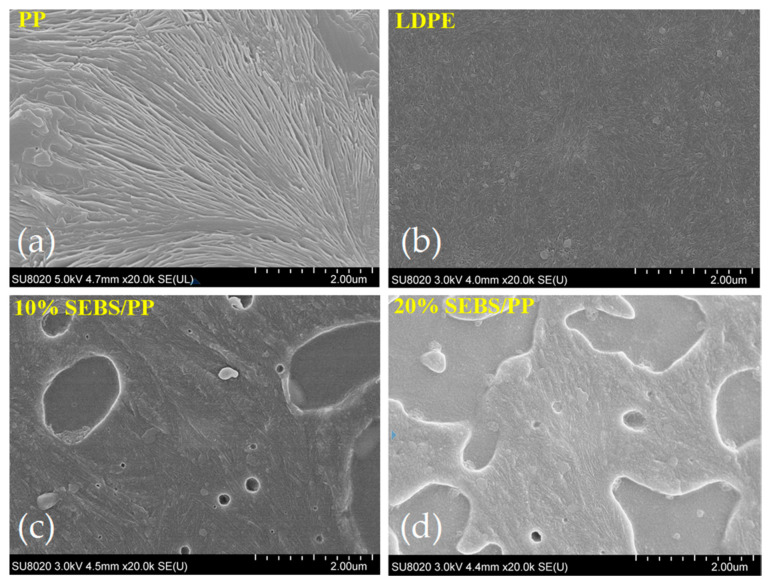
SEM images of lamella crystalline structures in (**a**) PP, (**b**) LDPE, (**c**) 10%SEBS/PP composite and (**d**) 10%SEBS/PP composite.

As shown by the crystallization morphologies of the PP, SEBS/PP composites and LDPE from SEM characterizations in Figure 3, the tightly stacked lamellae of the SEBS/PP composites are significantly larger than that of LDPE, which are supposed to become thinner and shorter for a higher SEBS content. The long and thick lamella do not appear in the 20%SEBS/PP composite, while the SEBS islands can be explicitly discriminated from the crystalline PP matrix. The similar alignments of lamellae in the pure and SEBS/PP composites imply that the SEBS additive can affect the PP macromolecular chains crystallizing into lamellae but will not change the formation mechanism of the PP spherulites.

**Figure 4:**
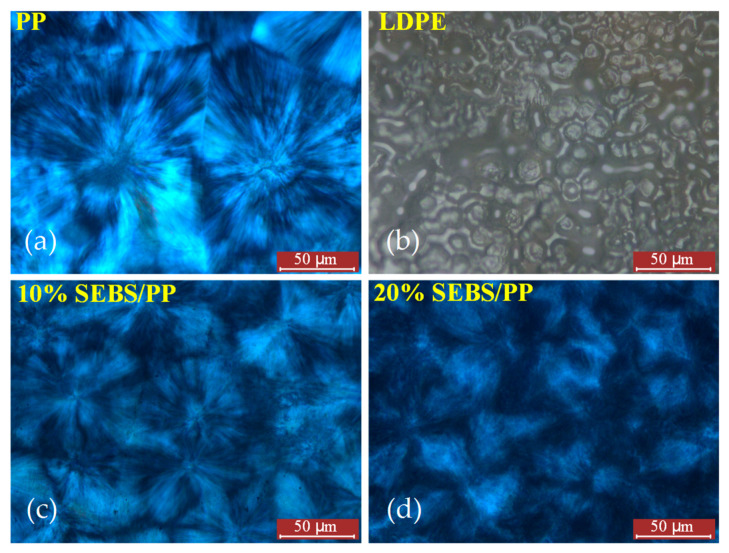
Spherulitic crystalline structures observed by the optical microscope for LDPE in normal mode and for PP and SEBS/PP composites in polarizing mode: (**a**) PP, (**b**) LDPE, (**c**) 10%SEBS/PP composite and (**d**) 10%SEBS/PP composite.

The optical microscopy photos illustrating the spherulite morphologies of the PP, SEBS/PP composites and LDPE are shown in Figure 4. The pure PP exhibits regular crystal structures of tightly arranged spherulites in clear polygons with diameters of 100~150 μm, which are remarkably larger than the ~4 μm of LDPE spherulites, resulting in a much smaller area of the amorphous phase than that of LDPE. In contrast, the PP spherulites become smaller, with the spherulite boundaries being obscured when SEBS is blended into PP, which is more obvious for a higher content of the SEBS elastomer. Especially, no obvious spherulite and boundary are shown in the 20%SEBS/PP composite. According to the crystallization kinetics, the lamellae are formed by the regular folding of macromolecular chains, while the spherulites consist of lamellae radically diverging from the spherulite nucleus. Therefore, SEBS can inhibit the formation of PP lamellae and, thus, will hinder the spherulite crystallization. Nevertheless, SEBS/PP composites still persist as a definitely higher spherulite size compared with LDPE.

**Figure 5:**
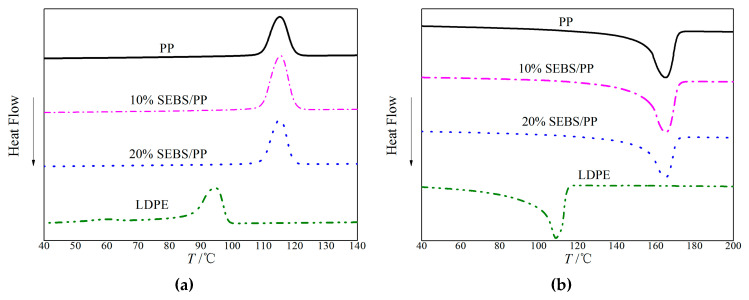
Differential scanning calorimetry (DSC) temperature spectra of the SEBS/PP composites and LDPE for the (**a**) crystallizing and (**b**) melting processes.

Figure 5 shows the melting-crystallization curves of PP, SEBS/PP composites and LDPE from differential scanning calorimetry (DSC) tests, with the characteristic results of the crystallization temperature *T*_c_, melting temperature *T*_m_ and crystallinity *X*_c_ being listed in Table 1. Compared with LDPE, SEBS/PP composites represent evidently higher *T*_m_ and *T*_c_, which are relevant to the intermolecular forces, chain compliance and molecular tacticity. It is well-known that the *T*_m_ will increase with the intermolecular forces and chain compliance. The methyl groups on the side chains of the PP molecules lead to a higher intermolecular force than the LDPE molecules without any substituent group. Furthermore, the steric hindrance of the PP macromolecular chains is higher than that of the LDPE molecules, which is against the internal rotation of the polymer chains and thereby results in a higher rigidity and a lower flexibility. More importantly, the PP macromolecular chains crystallize into a spiral molecular crystal, while the LDPE molecules condense into a full-return plane zigzag crystal, which means a higher temperature is required for relaxing the PP molecules than LDPE. Accordingly, the melting-crystallization curves show remarkably higher *T*_m_ and *T*_c_ of the PP than LDPE, even after blending SEBS, which is of great significance in engineering applications. However, the PP crystallinity decreases apparently after blending SEBS, which exists as amorphous structures in the SEBS/PP composites, approaching ~37.5% for the SEBS content of 20%.

### 2.3. Viscoelastic Properties

**Figure 6:**
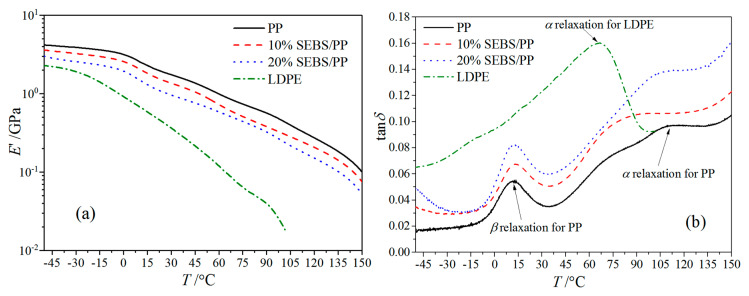
The dynamic thermomechanical analyzer (DMA) temperature spectra of the SEBS/PP composites and LDPE: (**a**) storage modulus and (**b**) loss factor.

An energy storage modulus as an effective elastic modulus for the polymers represents the material rigidity, while the value of the loss modulus directly characterizes the relaxation activities, as shown by the dynamic thermomechanical analyzer (DMA) results of the PP, LDPE and SEBS/PP composites in Figure 6. PP shows the highest storage modulus and rigidity, while the storage modulus of the SEBS/PP composite decreases gradually with the increase of the SEBS content. The storage moduli of the SEBS/PP composites and LDPE decrease with the increase of the temperature, which is due to exacerbation in the activity of the molecular chains caused by temperature elevation. Since the storage modulus is related to the general elastic deformation caused by changes in the bond length and bond angle of the molecular chains, the high crystallinity will generally lead to a higher storage modulus, accounting for the higher storage modulus of the SEBS/PP composites than that of LDPE [18]. The PP crystallinity decreases after blending the amorphous elastomer SEBS to form the island phase in the composites and is vulnerable to external forces, which results in a significant abatement in the storage modulus.

The loss factor tan*δ* peak at 10 °C indicates the glass transition temperatures of the PP matrix in the SEBS/PP composites. The loss factor peak at 80~110 °C is called the α relaxation peak, deriving from the relaxation movements of the molecular chains in the amorphous phase, which bears greater hindrance from the crystallization for higher crystallinity, thus requiring a higher temperature (molecular kinetic energy) to realize relaxation [19]. This accounts for the shifting of the PP α-peak towards a low temperature for the SEBS/PP composites, which is consistent with the crystallinity results, as shown in Table 1. Meanwhile, the higher crystallinity leads to the greater thickness of the lamellae and results in a higher relaxation, which can also consistently be verified by Figure 3 and Figure 5b. The PP materials have a higher crystallization compared with LDPE, leading to an abatement in the molecular relaxations of the amorphous regions between the lamellae, as indicated by the lower α-peak than LDPE. It is consistently shown from the crystallization morphologies of Figure 3 and Figure 4 that the PP spherulite structure becomes looser with the lamellae being decreased in size after the addition of SEBS, which increases the intensity of the α relaxation peak, as shown in Figure 6a.

### 2.4. Stress-Strain Characteristics

**Figure 7:**
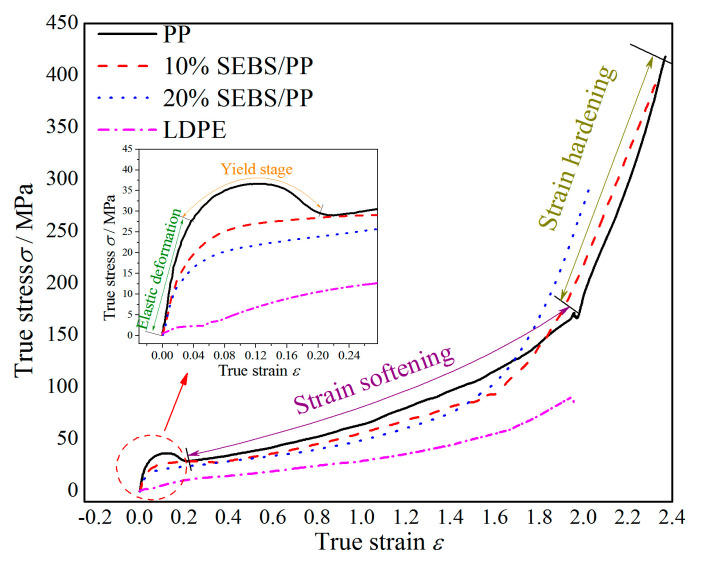
Stress-strain characteristics of the SEBS/PP composites and LDPE.

The stress-strain characteristics, as shown in Figure 7, indicate that the elastic modulus, yield stress, fracture stress and elongation at break of SEBS/PP are much higher than those of LDPE; the yield stress of the SEBS/PP composite decreases gradually with the increase of the SEBS content, retaining a significant higher yield stress than that of LDPE as the SEBS content is raised to 20%; the stress of the PP and SEBS/PP composites increase significantly with the increase of the strain during the strain-strengthening stage, showing an observable feature of strain-hardening.

According to the molecular dynamics theory, the material fractures originate essentially from the micromovements of molecular chains. At each stage of the stress-strain characteristics, the microstructures of the crystal and amorphous regions, which bear different stresses, evolve in discrepant forms. At the elastic stage, the essential deformation is attributed to the variations in the bond length and bond angle of the molecular chains; in the yielding process, the ability of the semi-crystalline polymer material undertaking an external force is mainly contributed by the irregularly connected molecular chains between the lamellae; at the strain-softening stage, the relative slip between the folded chains occurs in the crystal region, and the unwinding behavior occurs in the amorphous region on a larger scale; as the strain continues to increase, strain-hardening arises from the restriction of the connecting molecular chains in the amorphous phase, until the connecting molecular chains cannot elongate further and fracture under external forces.

The PP material represents a higher crystallization ability and larger sizes of spherulite and lamellae compared with LDPE, which enhances the binding force of the crystal phase to the amorphous phase between lamellae, resulting in a much higher yield strength of the SEBS/PP composites than that of LDPE. Since the elastomer SEBS is prone to deformation under tensile stress, the PP lamellae in the SEBS/PP composites are thinner and shorter compared with pure PP, which leads to the decrease of the yield strength as a manifestation of the mechanical degradation in the amorphous phase for bearing stress. It is also noted from Figure 7 that the PP and SEBS/PP show a “strain-hardening” phenomenon more obviously than pure LDPE, which is attributed to the restriction of connecting molecular chains in the amorphous phase. At the strain-hardening stage, the molecular chains connecting lamellae cannot further elongate under the action of external force and begin unwrapping even to be broken, leading to the gradual disintegration of polyethylene crystallization. It needs a large stress to be break the high regularity of crystallization, as illustrated by the sharply increased stress of the specimen with the increase of the strain in the strain-hardening stage. Furthermore, the more obvious strain-hardening phenomenon appears accompanied with a higher crystallinity. In combination with the evidently higher crystallinities of PP and SEBS/PP than that of LDPE, it can be consistently derived that PP and SEBS/PP will bear more strain-hardening processes than LDPE. The polymer materials with strain hardening generally have high fracture stress and elongation at breaks, so the higher strain hardening means that the material can withstand larger stress and strain.

### 2.5. Water-Tree Resistance Performance

**Figure 8:**
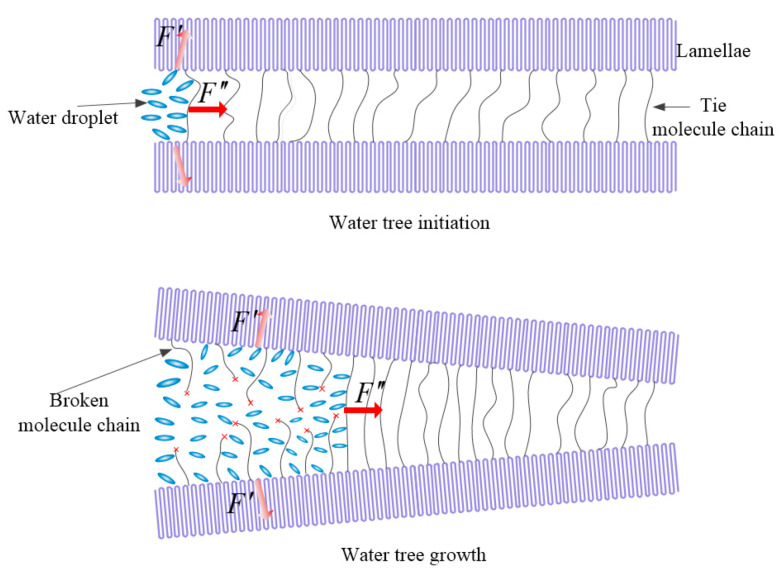
Schematic electromechanical mechanism of water-tree initiation (above panel) and growth (bottom panel) in SEBS/PP composites. *F*′ and *F*″ in represent the impact forces of water microbeads on the lamellae and the molecular chains connecting adjacent lamellae, respectively.

Although no unified conclusion has been reached on the mechanism of water-tree aging, the electromechanical degradation mechanism of water-tree growth is considered as the most preferable recognition [10,20]. High electric field aggregating at the microdefects in dielectric materials can generate a dipole moment in micro-water beads so that the pulsating stress along the direction of the electric field gradient will be produced under the divergent electric field (electrophoretic force). Due to the perfect structure and the stable physical and chemical properties of the crystalline phase, the pulsating stress of micro-water beads inclines to destroy the amorphous phase, as shown in Figure 8, illustrating that cracks are engendered around a part of the amorphous phase that has been completely penetrated by microbeads, from where water molecules are injected into these cracks to subsequently form micro-water beads for developing eventually into water trees. The *F*′ and *F*″ in Figure 8 represent the impact forces of water microbeads on the lamellae and the molecular chains connecting adjacent lamellae, respectively. After a long period of *F*′ and *F*″ actions, the PP crystal will be separated completely, and the connecting molecular chain between lamellae will eventually break, which provides the requisite space for the growth of water trees.

The electromechanical degradation mechanisms of polymer water-tree initiation and growth indicate two necessary conditions required for water-tree formation: (1) the amorphous phase between lamellae providing growth channels for water trees and (2) that micro-water beads can enter the amorphous phase to form water-filled holes under an AC electric field. PP molecules can efficiently crystallize into a large spherulite with the thick and long lamellae being tightly stacked, resulting in narrow amorphous regions between lamellae that will not allow water-tree growth. The macroscopic mechanical properties of PP such as a large storage modulus, strong rigidity and high yielding strength imply that the order arrangement of the polymer chains in crystallization impedes the molecular relaxations in the amorphous phase between the crystalline structures, which is manifested by a low α relaxation peak and indicates that pulsating water droplets polarized under an AC electric field are incompetent for causing relative slips between lamellae and making an effective channel for water-tree growth.

In contrast to PP and LDPE, the amorphous SEBS is a thermoplastic elastomer that can alleviate the centralization of external forces at the structural defects. The “islands” formed by amorphous SEBS in the composites are very probable to provide transport tunnels for water molecules and, thus, expedite water-tree growth between lamellae. The higher α relaxation peak and the lower material yield strength of the SEBS/PP composites than that of pure PP present a manifestation that the activity of the amorphous regions and the relative slips between lamellae have been promoted by introducing SEBS into the PP matrix. It is noted from the crystalline morphology and melting crystallization characteristics that SEBS/PP composites build a larger size of lamellae and spherulites, which will cause a higher restriction on the amorphous regions.

## 3. Material Preparation and Testing Method

### 3.1. Material Preparation

Raw materials of iPP (NT30s, China Petrochemical Corporation, Shanghai, China) and SEBS (G1652EU, Kraton Polymers Corporation, Houston, TX, USA) are utilized, respectively, as resin matrix and elastomer additive to prepare SEBS/PP composites as follows: The PP and SEBS and LDPE are first placed in a vacuum environment for 24 h at a temperature of 80 °C to fully remove moisture from the raw materials and then completely mixed for 15 min in a torque rheometer (Harbin Hapro Electric Technology Co. Ltd., Harbin, China) at a temperature of 200 °C to obtain SEBS/PP composites individually with the SEBS contents of 10% and 20% (respectively denoted by 10%SEBS/PP and 20%SEBS/PP). The PP, SEBS/PP and LDPE materials are pressed by the melt-molding method into the shape required for the subsequent experiments as follows [21]: The three kinds of prepared materials are firstly put into a plate vulcanizing machine (XLB25-D, Huzhou Xingli Rubber Machinery Manufacturing Co. Ltd., Hangzhou, China) for 10 min at 200 °C temperature without pressure; after, the material is fully melted, and the pressure of plate vulcanizer is increased to 15 MPa maintained for 15 min so that the pressed samples are obtained in a required shape; the pressed samples are finally cooled for 3 min under a pressure of 15 MPa at the temperature of 15 °C in the plate vulcanizer to complete the material sample preparations.

### 3.2. Accelerated Water-Tree Aging Experiment

The accelerated water-tree aging experiments are performed by means of water blade electrodes which can efficiently produce water-trees in a clear morphology [22]. The knife-like defect is caused at the edge of blade electrode by cutting the conductive metal blade with a curvature radius of 0.01 mm and a thickness of 0.03 mm vertically into the side surface of the cuboid sample in a length of 100 mm and a thickness of 4 mm, a distance of 2 mm being kept from the blade edge to the other side of cuboid surface. The water medium of 1.8 mol/L sodium chloride solution is used for water-tree aging experiments. To increase the initiation probability and growth rate of water-trees with appreciable morphology characteristics for different materials, the AC high voltage power supply with a frequency of 3 kHz and an effective value of 4 kV is adopted to continuously apply on samples for 7 days. Before applying voltage, the whole equipment for water-tree experiments is placed in a vacuum environment being kept for 30 min to fully eliminate the residual air in blade edge defects.When water-tree experiments of applying voltage is finished, the defects at blade edge are cut along longitudinal direction of cuboid sample into thin slices with a thickness of 120 μm, which are impregnated in methylene blue solution for 4 h at the temperature of 90 °C.

### 3.3. Crystalline Morphology Observation

The water trees produced in the prepared slice samples with a thickness of 120 μm are preliminarily observed for crystalline morphology by a polarizing fluorescence microscope (PLM, DM2500P, Leica Co., Berlin, Germany). The lamella crystal morphology and the SEBS dispersion in the composites are characterized with an ultra-high-resolution cold field emission scanning electron microscope (SEM, SU8020, Hitachi Co., Tokyo, Japan) with a magnification of 2000 under the accelerating voltage of 0.5 kV~30 kV. Slice samples with a thickness of 1mm are brittlely fractured in liquid nitrogen at a low temperature and then immersed in the etching solution of permanganic acid for 7 h to observe the crystalline structures of the PP, SEBS/PP composites and LDPE, in which the etching solution is used to preferentially etch the amorphous part so that the crystalline morphology appears clearly [23]. Subsequently, the fractured slice samples with etched surfaces are washed in an ultrasonic cleaning machine (Harbin Hapro Electric Technology Co. Ltd., Harbin, China) with a detergent composed of concentrated sulfuric acid, water and hydrogen peroxide in a volume ratio of 2:7:1. Finally, a very thin gold layer is sputtered onto the fractured surface to prevent any undesirable charges from accumulating on the sample surface during SEM characterizations.

### 3.4. Thermomechanical Tests

Employing the differential scanning calorimetry (DSC) method, the heat-flow temperature thermograms in the crystallizing and melting processes are tested with a thermal analyzer (DSC-3, METTLER TOLEDO, Zurich, Switzerland) under nitrogen atmosphere in the temperature range of 40~200 °C with a heating/cooling rate of 10 °C/min. The crystallinity is calculated by *X*_c_ = Δ*H*_m_/Δ*H*_100_, in which Δ*H*_m_ and Δ*H*_100_ denote the melting enthalpies of the tested sample and the ideal crystal material in 100% crystallinity (207 J/g for PP and 288 J/g for LDPE), respectively.

According to the dynamic mechanics analysis (DMA) method, the material viscoelasticity of the samples in dimensions of 80 × 10 × 1 mm is evaluated by measuring the energy-storage modulus *E*’ and loss factor tan*δ* in the temperature range of −50~150 °C, as implemented by a dynamic thermomechanical analyzer (Q800DMA, TA apparatus Co. Ltd., New Castle, DE, USA), with a heating rate of 3 °C/min in nitrogen atmosphere. The DMA tests are implemented under the tensile mode, with the target amplitude, frequency, static force and dynamic force being set as 15 μm, 1 Hz, 0.375 and 0.3 N, respectively. The stress-strain characteristics are tested conforming to the GB/T 1040.2-2006 standard with an elongation speed of 5 mm/min for the samples being fabricated into a “5 A” dumbbell shape in a thickness of 1mm and a mark distance of 20 mm.

## 4. Conclusions

Water-tree aging resistances of PP, SEBS/PP composites and LDPE are comparatively investigated with the water-blade electrode method. The water-tree morphology and crystallization characteristics are observed in coordination with mechanical tests of the viscoelasticity and stress-strain process so as to elucidate the water-tree growth mechanism that is essentially correlated with crystallization morphology. Both the highly crystalline PP and the amorphous elastomer SEBS show extremely high resistances to water trees, while the SEBS/PP composites exhibit substantial water-tree aging, in which the amorphous SEBS exists as an “island” phase in the crystalline PP matrix. The addition of SEBS has little effect on the crystallinity and crystallization melting temperature of the PP phase but makes the PP spherulite boundary become blurred and results in shorter and thinner lamellae so as to create available void space for water-tree growth in amorphous regions between lamellae. The presence of SEBS facilitates the relative slips between lamellae and, thus, makes it easier for micro-water beads to destroy the amorphous regions in the PP matrix to form water-tree propagation channel materials under a divergent electric field. The better water-tree resistance performances of the SEBS/PP composites than that of LDPE can be consistently attributed to their lower loss factor peak and higher yielding strength derived from the crystalline morphology with a higher density of lamellae stacking.

## Figures and Tables

**Table 1:** Crystallization temperature *T*_c_, melting temperature *T*_m_ and crystallinity *X*_c_ of PP, LDPE and SEBS/PP composites.

Material Samples	*T*_m_/°C	*T*_c_/°C	*X*_c_/%
PP	166.3	115.1	46.2
10%SEBS/PP	166.5	113.2	40.8
20%SEBS/PP	166.3	115.5	37.5
LDPE	104.0	94.9	33.6
